# Supplementary material for: Circadian Variation in Human Milk Composition, a Systematic Review
Source: Nutrients. 2020 Aug 4;12(8):2328. doi: 10.3390/nu12082328 (PMC7468880; doi:10.3390/nu12082328)
Supplement: Supplementary file 1 [file nutrients-12-02328-s001.zip › Supplemental File S1_Literature Search.docx]

Supplemental File S1: Literature Search

**Search strategy: Circadian variation in human milk composition**

**Embase.com**

('periodicity'/exp OR (photoperiod* OR ((activit*) NEAR/3 (cycle*)) OR ((period* OR day* OR 24 OR 24h OR twenty-four OR twentyfour OR electrolyt* OR nutrient* OR micronutrient* OR macronutrient* OR immun* OR hormon* OR endocrine* OR trace-element* OR mineral* OR vitamin* OR carbohydrat* OR fat OR fats OR fatty OR protein* OR amino-acid* OR cortisol* OR cortison* OR oligosaccharide* OR melatonin* OR probiotic* OR microbiota) NEAR/3 (variat* OR fluctuat* OR change* OR recur* OR cycle*)) OR ((bio*) NEAR/3 (clock*)) OR rhythm* OR circadian* OR diurnal* OR biorhythm* OR ultradian*):ab,ti,kw) AND ('breast milk'/exp OR (breastmilk* OR breastfeed* OR ((breast* OR human* OR maternal* OR mother* OR woman*) NEAR/3 (milk*)) OR ((breast*) NEAR/3 (feed*))):ab,ti,kw) NOT ([animals]/lim NOT [humans]/lim)

**Medline (Ovid)**

(exp Periodicity/ OR (photoperiod* OR ((activit*) ADJ3 (cycle*)) OR ((period* OR day* OR 24 OR 24h OR twenty-four OR twentyfour OR electrolyt* OR nutrient* OR micronutrient* OR macronutrient* OR immun* OR hormon* OR endocrine* OR trace-element* OR mineral* OR vitamin* OR carbohydrat* OR fat OR fats OR fatty OR protein* OR amino-acid* OR cortisol* OR cortison* OR oligosaccharide* OR melatonin* OR probiotic* OR microbiota) ADJ3 (variat* OR fluctuat* OR change* OR recur* OR cycle*)) OR ((bio*) ADJ3 (clock*)) OR rhythm* OR circadian* OR diurnal* OR biorhythm* OR ultradian*).ab,ti,kw.) AND (Milk, Human/ OR (breastmilk* OR breastfeed* OR ((breast* OR human* OR maternal* OR mother* OR woman*) ADJ3 (milk*)) OR ((breast*) ADJ3 (feed*))).ab,ti,kw.) NOT (exp animals/ NOT humans/)

**Web of Science**

TS=(((photoperiod* OR ((activit*) NEAR/2 (cycle*)) OR ((period* OR day* OR 24 OR 24h OR twenty-four OR twentyfour OR electrolyt* OR nutrient* OR micronutrient* OR macronutrient* OR immun* OR hormon* OR endocrine* OR trace-element* OR mineral* OR vitamin* OR carbohydrat* OR fat OR fats OR fatty OR protein* OR amino-acid* OR cortisol* OR cortison* OR oligosaccharide* OR melatonin* OR probiotic* OR microbiota) NEAR/2 (variat* OR fluctuat* OR change* OR recur* OR cycle*)) OR ((bio*) NEAR/2 (clock*)) OR rhythm* OR circadian* OR diurnal* OR biorhythm* OR ultradian*)) AND ((breastmilk* OR breastfeed* OR ((breast* OR human* OR maternal* OR mother* OR woman*) NEAR/2 (milk*)) OR ((breast*) NEAR/2 (feed*))))) **Cochrane CENTRAL**

((photoperiod* OR ((activit*) NEAR/3 (cycle*)) OR ((period* OR day* OR 24 OR 24h OR twenty-four OR twentyfour OR electrolyt* OR nutrient* OR micronutrient* OR macronutrient* OR immun* OR hormon* OR endocrine* OR trace-element* OR mineral* OR vitamin* OR carbohydrat* OR fat OR fats OR fatty OR protein* OR amino-acid* OR cortisol* OR cortison* OR oligosaccharide* OR melatonin* OR probiotic* OR microbiota) NEAR/3 (variat* OR fluctuat* OR change* OR recur* OR cycle*)) OR ((bio*) NEAR/3 (clock*)) OR rhythm* OR circadian* OR diurnal* OR biorhythm* OR ultradian*):ab,ti,kw) AND ((breastmilk* OR breastfeed* OR ((breast* OR human* OR maternal* OR mother* OR woman*) NEAR/3 (milk*)) OR ((breast*) NEAR/3 (feed*))):ab,ti,kw)

**Google Scholar**

Photoperiod|periodicity|"period|day|24|24h|electrolytes|nutrients|hormones|minerals|vitamins|carbohydrates|fats|fatty|proteins variation|fluctuation|changes|cycle"|"biological clock"|rhythm|circadian breastmilk|breastfeeding|"breast|human|maternal milk"
