# Supplementary material for: Circadian Variation in Human Milk Composition, a Systematic Review
Source: Nutrients. 2020 Aug 4;12(8):2328. doi: 10.3390/nu12082328 (PMC7468880; doi:10.3390/nu12082328)
Supplement: Supplementary file 1 [file nutrients-12-02328-s001.zip › Supplemental File S3_Results of modified NOS for cross-sectional studies.docx]

Supplemental File S3: Results of modified Newcastle Ottawa grading Scale (NOS)
