# Supplementary material for: Circadian Variation in Human Milk Composition, a Systematic Review
Source: Nutrients. 2020 Aug 4;12(8):2328. doi: 10.3390/nu12082328 (PMC7468880; doi:10.3390/nu12082328)
Supplement: Supplementary file 1 [file nutrients-12-02328-s001.zip › Supplemental File S4_Circadian variation in human milk composition.docx]

Supplemental File S4: Circadian variation in human milk composition

***Macronutrients***

|  | Year | Location | N | Maternal  characteristics | Gestational  age at birth | Milk type | Postpartum   period | Collection   days | Collection  timepoints  (hours) | Analytical   method | RoB score | Circadian   pattern? |
| --- | --- | --- | --- | --- | --- | --- | --- | --- | --- | --- | --- | --- |
| **Carbohydrates** | | | | | | | | | | | | |
| Carbo-hydrates | 2017 [23] | Turkey | 52 | CS (n=33) +  VD (n=19) | P+T | F+H | >5d |  | 6-9, 12-15, 21-00 | IRS | 7 | no |
|  | 2019 [24] | NL | 4 |  | T (37-42w) | F | 30d (+/- 5d) | 1 | 5x in 24 hours | Mid IRS | 6 | no |
|  | 2015 [25] | Israel | 32 |  | P (25-35w) | Mix | 1w, 2w, 3w, 4w, 5w, 6w, 7w | 7x1 | 6-9, 21-00 | Mid IRS | 6 | no |
| Glucose | 1991 [26] |  | 12 |  |  | H | 1-13m | 1 | 8, 12, 16, 20, 24, 4 | EA | 6 | yes |
|  | 1990 [27] |  | 6 |  | T | H | 8w | 1 | 6, 10, 14, 18, 22 | EA | 7 | no |
| G6P, G1P | 1991 [26] |  | 12 |  |  | H | 1-13m | 1 | 8, 12, 16, 20, 24, 4 | EA | 6 | no |
| UDP-glucose | 1991 [26] |  | 12 |  |  | H | 1-13m | 1 | 8, 12, 16, 20, 24, 4 | EA | 6 | no |
| Lactose | 1991 [26] |  | 12 |  |  | H | 1-13m | 1 | 8, 12, 16, 20, 24, 4 | Glucose oxidase method | 6 | no |
|  | 2015 [28] | Australia | 19 | PP + MP | T | F+H | 3-21w | 1 | 4-10, 10-16, 16-22, 22-4 | SP | 8 | yes |
|  | 2013 [29] | Australia | 15 |  | T | F+H | 1-6m | 1 | 4-10, 10-16, 16-22, 22-4 | SP | 6 | no |
|  | 1990 [27] |  | 6 |  | T | H | 8w | 1 | 6, 10, 14, 18, 22 | EA | 7 | no |
|  | 1986 [30] | France | 5 | PP + MP |  |  | 0-7d | 1 |  |  | 5 | no |
| UDP-galactose | 1991 [26] |  | 12 |  |  | H | 1-13m | 1 | 8, 12, 16, 20, 24, 4 | EA | 6 | no |
|  | Year | Location | N | Maternal  characteristics | Gestational  age at birth | Milk type | Postpartum   period | Collection   days | Collection  timepoints  (hours) | Analytical   method | RoB score | Circadian   pattern? |
| **Proteins** | | | | | | | | | | | | |
| Total protein | 2015 [28] | Australia | 19 | PP + MP |  | F+H | 3-21w | 1 | 4-10, 10-16, 16-22, 22-4 | EA | 8 | no |
|  | 2017 [23] | Turkey | 52 | CS (n=33) +  VD (n=19) | P+T | F+H | >5d |  | 6-9, 12-15, 21-00 | IRS | 7 | no |
|  | 1979 [32] | UK | 1 | PP + MP |  | F+H | 5-42w | 3 | 6, 10, 14, 18 | SP | 3 | no |
|  | 2019 [24] | NL | 4 |  | T (37-42w) | F | 30d (+/- 5dgn) | 1 | 5x in 24 hours | Mid IRS | 6 | no |
| *Skim milk protein* | 2012 [34] | Australia | 15 | PP + MP | T, singletons | F+H | 1-6m | 1 | 4-10, 10-16, 16-22, 22-4 | EP | 6 | no |
| *Whey protein* |  |  |  |  |  |  |  |  |  | EP |  | no |
| *Casein protein* |  |  |  |  |  |  |  |  |  | EP |  | no |
|  | 2013 [29] | Australia | 15 |  | T | F+H | 1-6m | 1 | 4-10, 10-16, 16-22, 22-4 | Bradford assay | 6 | no |
|  | 1990 [27] |  | 6 |  | T | H | 8w | 1 | 6, 10, 14, 18, 22 | KM | 7 | yes |
|  | 2013 [35] | Brazil | 23 | Normo-tensive (n=10), hyper-tensive (n=13) |  |  | 3d, 10d, 23d | 3x1 | 12,24 | SP | 5 | no |
|  | 2015 [25] | Israel | 32 |  | P (25-35w) | Mix | 1w, 2w, 3w, 4w, 5w, 6w, 7w | 7x1 | 6-9, 21-00 | Mid IRS | 6 | no |
|  | 2011 [36] | Spain | 77 |  |  | F | <5d, 6-15d, >15d | 3x1 | 8-20, 20-8 | KM | 6 | Yes, M |
|  | 1976 [33] | USA (Indiana) | 5 | PP + MP |  | F | 3w-3.5m | 2 | >4x in 24 hours | Microbiological assay | 9 | no |
| DSIP | 1984 [39] |  | 2 | PP |  | F+H | 0d-cessation | 30 (n=1), 25-cessation (n=1) | Morning, evening | RIA | 5 | yes, n=1 (mother sampling 0d-1m) |
| **Amino acids** | | | | | | | | | | | | |
| Amino acids |  |  |  |  |  |  |  |  |  |  |  |  |
| *Taurine* | 1987 [37] | USA (Connecticut) | 7 |  | T | F | 8w | 1 | 6, 10, 14, 18, 22 | HPLC reversed phase | 5 | No |
| *Glutamine + glutamate* |  |  |  |  |  |  |  |  |  | HPLC |  | No |
| *Tryptophan* | 2013 [43] | Spain | 77 |  |  | F | <5d, 6-15d, >15d | 3x1 | 8x in 24 hours | HPLC-MS/MS | 9 | Yes |
| *Methionine* |  |  |  |  |  |  |  |  |  | HPLC-MS/MS |  | yes, T + M |
| *Aspartic acid* |  |  |  |  |  |  |  |  |  | HPLC-MS/MS |  | yes, M |
| *Histidine* |  |  |  |  |  |  |  |  |  | HPLC-MS/MS |  | yes, M |
| *Phenylalanine* |  |  |  |  |  |  |  |  |  | HPLC-MS/MS |  | yes, M |
| *Tyrosine* |  |  |  |  |  |  |  |  |  | HPLC-MS/MS |  | yes, M |
| *Tryptophan* | 2005 [18] |  | 8 |  |  |  | 12w | 1 | 00-12, 12-00 | HPLC | 7 | yes |
| Nitrogen content | 1987 [37] | USA (Connecticut) | 7 |  | T | F | 8w | 1 | 6, 10, 14, 18, 22 | Micro KM | 5 |  |
|  | 1986 [38] |  | 6 |  | T | F+mid+H | 8w | 1 | 6, 10, 14, 18, 22 | KM | 5 | no |
|  | 2011 [36] | Spain | 77 |  |  | F | <5d, 6-15d, >15d | 3x1 | 8-20, 20-8 | KM | 6 | yes, M |
| Urea | 1987 [37] | USA (Connecticut) | 7 |  | T | F | 8w | 1 | 6, 10, 14, 18, 22 | SP | 5 | no |
| **Enzymes** | | | | | | | | | | | | |
| BSSL | 1986 [40] | USA (Colorado + Washington) | 12 |  | P+T (29-40w) |  | 4d-4m | 1 | 0-8, 8-16, 16-0 | EA | 5 | no |
|  | 1986 [38] |  | 6 |  | T | F+mid+H | 8w | 1 | 6, 10, 14, 18, 22 | EA | 5 | no |
| SSL | 1986 [38] |  | 6 |  | T | F+mid+H | 8w | 1 | 6, 10, 14, 18, 22 | EA | 5 | yes |
| LPL | 1986 [40] | USA (Colorado + Washington) | 12 |  | P+T (29-40w) |  | 4d-4m | 1 | 0-8, 8-16, 16-0 | EA | 5 | yes |
| Superoxide dismutase | 2016 [41] | Germany | 21 |  | P (27.6-36.1w) + T (37.0-39.4w) | H | 5-10d | 1 | 10-22, 22-10 | ELISA | 5 | no |
| Glutathion peroxidase | 2016 [41] | Germany | 21 |  | P (27.6-36.1w) + T (37.0-39.4w) | H | 5-10d | 1 | 10-22, 22-10 | ELISA | 5 | yes |
| Amylase | 1984 [42] |  | 1 |  |  | Mid | 7d | 1 | 5x in 24 hours | Phadebas amylase test | 2 | no |
|  | Year | Location | N | Maternal  characteristics | Gestational  age at birth | Milk type | Postpartum   period | Collection   days | Collection  timepoints  (hours) | Analytical   method | RoB score | Circadian   pattern? |
| **Fats** | | | | | | | | | | | | |
| Total fat | 2017 [23] | Turkije | 52 | CS (n=33) +  VD (n=19),  PP + MP | P+T | F+H | >5d |  | 6-9, 12-15, 21-00 | IRS | 7 | no |
|  | 2010 [54] | Brazilië | 36 |  | T |  | 3d, 10d, 30d | 3x1 | 12, 00 | CM | 5 | no |
|  | 1949 [44] |  | 4 |  |  | F | 8-11d | 52u | 4, 8, 12, 16, 20, 00 | Gerber method | 3 | yes |
|  | 1979 [32] | UK | 1 |  |  | F+H | 5-42w | 3 | 6, 10, 14, 18 | Gravimetric analysis | 3 | yes |
|  | 2019 [24] | NL | 4 |  | T (37-42w) | F | 30d (+/- 5d) | 1 | 5x in 24 hours | Mid IRS | 6 | yes |
|  | 1988 [45] | Thailand | 19 | PP + MP | T | F+H | <9m | 1 | 8-12, 12-16, 16-20, 20-00 | CM | 4 | yes |
|  | 1995 [46] |  | 10 | Insulin dependent DM (n=5), no IDDM (n=5), PP + MP |  | F + mid + H | 84d | 1 | 6-10, 10-14, 14-18, 18-22, 22-02, 02-6 | Gravimetric analysis | 7 | yes |
|  | 2013 [29] | Australia | 15 |  | T | F+H | 1-6m | 1 | 4-10, 10-16, 16-22, 22-4 | CM | 6 | yes |
|  | 2012 [47] | Poland | 61 |  | P (26-36w, n=22) + T (>37w, n=39) |  |  | 3 | 6-9, 0-3 | CM | 6 | yes, C + M |
|  | 1990 [27] |  | 6 |  | T | H | 8w | 1 | 6, 10, 14, 18, 22 | Gravimetric analysis | 7 | no |
|  | 1986 [38] |  | 6 |  | T | F+mid+H | 8w | 1 | 6, 10, 14, 18, 22 | Folch method | 5 | yes |
|  | 2006 [48] |  | 39 |  | P (26-33w) | F+mid+H | 7-14d | 1 | 6-9, 21-00 | CM | 4 | yes |
|  | 2007 [49] |  | 22 |  | P (26-31w) | F+mid+H | 2w, 3w, 4w, 5w, 6w, 7w | 6x1 | 6-9, 21-00 | CM | 4 | yes |
|  | 1978 [50] | Canada | 2 |  |  | Mix |  | 1 | Morning, midday, evening | GC | 3 | yes, only in donor 2 |
|  | 2015 [25] | Israel | 32 |  | P (25-35w) | Mix | 1w, 2w, 3w, 4w, 5w, 6w, 7w | 7x1 | 6-9, 21-24 | EA | 6 | yes |
|  | 1976 [51] | USA | 25 | PP + MP | P + T | F | 6-12w | 1 |  |  | 7 | yes |
|  | 1981 [111] | UK | 13 | P + T |  | F+H+mix | 3-10d, >10d | 2x1 |  |  | 4 | no |
|  | 1994 [52] | Mexico | 10 | PP + MP |  | Mix |  | 1 | 4x/24 hours | Folch method | 7 | yes |
|  | 2006 [53] |  | 71 | PP + MP | T | F+H | 1-6m | 1 | 4-10, 10-16, 16-22, 22-4 | CM | 5 | yes |
| Fat globules | 1988 [63] | Denmark | 5 |  |  |  | >7d | 1 | 10, 22 | EA | 6 | yes |
| Triglyce- rides | 1986 [40] | USA (Colorado + Washington) | 12 |  | P+T (29-40w) |  | 4d-4m | 1 | 0-8, 8-16, 16-0 | EA | 5 | no |
|  | 1983 [55] | UK, Germany | 7 |  | T | F | 15d, 22d, 29d, 36d | 4 (pooled) | 00-6, 6-12, 12-18, 18-00 | EA | 5 | yes |
|  | 1997 [57] | Guatamala | 52 |  |  |  | 1-4m (mean 2.8±1.1) | 1-2 | 6x/24 hours (1 night sample) | Optical density | 8 | yes |
| Fatty acids | 1977 [59] |  | 8 |  |  | F | 6-12w | 1 | Morning, midday, evening | GC | 4 | no |
|  | 1979 [32] | UK | 1 | PP + MP |  | F+H | 5-42w | 3 | 6, 10, 14, 18 | Gravimetric analysis | 3 | no |
|  | 1983 [55] | UK, Germany | 7 |  | T | F | 15d, 22d, 29d, 36d | 4 (pooled) | 00-6, 6-12, 12-18, 18-00 | GC | 5 | no |
|  | 1990 [27] |  | 6 |  | T | H | 8w | 1 | 6, 10, 14, 18, 22 | GC | 7 | no |
|  | 2003 [60] | Australia | 5 | MP |  | H | 1m, 2m, 4m, 6m, 9m, 12m | 6x1 |  | GC | 5 | no |
|  | 2002 [61] | Australia | 17 | PP + MP | T | F+H | 4m, 6m | 2x1 |  | Colorimetric SM | 6 | no |
| Sphingo- myelin | 1986 [62] | USA (boston) | 5 |  |  | F+H | 39-85d | 72h | Each feeding for 72 hours | RIA-like | 8 | no |
| Cholesterol | 1983 [55] | UK, Germany | 7 |  | T | F | 15d, 22d, 29d, 36d | 4 (pooled) | 00-6, 6-12, 12-18, 18-00 | EA | 5 | Yes, only in English mothers |
|  | 1995 [46] |  | 10 | Insulin dependent DM (n=5), no IDDM (n=5), PP + MP |  | F+mid+H | 84d | 1 | 6-10, 10-14, 14-18, 18-22, 22-02, 02-6 | TLC | 7 | yes |
|  | 1990 [27] |  | 6 |  | T | H | 8w | 1 | 6, 10, 14, 18, 22 | GC | 7 | no |
|  | 1978 [58] | USA | 22 |  |  | F | 6-12w | 1 (n=18); 5 (n=4) | 3-6x in 24 hours | Colorimetric SM | 7 | yes |
| Phospho- lipids | 1983 [55] | UK, Germany | 7 |  | T | F | 15d, 22d, 29d, 36d | 4 (pooled) | 00-6, 6-12, 12-18, 18-00 | TLC | 5 | yes |

***Micronutrients***

|  | Year | Location | N | Maternal  characteristics | Gestational  age at birth | Milk type | Postpartum   period | Collection   days | Collection  timepoints  (hours) | Analytical   method | RoB score | Circadian   pattern? |
| --- | --- | --- | --- | --- | --- | --- | --- | --- | --- | --- | --- | --- |
| **Vitamins** | | | | | | | | | | | | |
| Vitamin A | 2017 [64] | Bangladesh | 18 |  |  | F+mid+H | 2-4m | 1 | 12 intervals in 24 hours | HPLC + LC-MS | 8 | no |
| Vitamin B |  |  |  |  |  |  |  |  |  |  |  |  |
| *Vit B1* | 2017 [64] | Bangladesh | 18 |  |  | F+mid+H | 2-4m | 1 | 12 intervals in 24 hours | HPLC + LC-MS | 8 | no |
| *Vit B2* |  |  |  |  |  |  |  |  |  | HPLC + LC-MS |  | no |
| *Vit B3* |  |  |  |  |  |  |  |  |  | HPLC + LC-MS |  | no |
| *Vit B6* |  |  |  |  |  |  |  |  |  | HPLC + LC-MS |  | no |
| *Vit B12* |  |  |  |  |  |  |  |  |  | HPLC + LC-MS |  | no |
| *Folate + folic acid* | 1987 [68] | USA (Indiana) | 27 | PP + MP | T (37-40w) | H | 2w, 1m, 2m, 3m, 4m, 5m, 6m, 8m, 10m, 12m | 10x1 | 12 intervals in 24 hours | Microbiological assay | 7 | yes |
| *Vit B6* | 1976 [33] | USA (Indiana) | 5 | PP + MP |  | F | 3w-3.5m | 2 |  | Microbiological assay | 9 | no |
| *Vit B8 (biotine)* | 1992 [65] | USA (Iowa) | 5 | PP + MP |  | Mid | >18d | 1 | On demand breastfeeding | I- Avidin assay | 3 | no |
| Vitamin E |  |  |  |  |  |  |  |  |  |  |  |  |
|  | 2017 [64] | Bangladesh | 18 |  |  | F+mid+H | 2-4m | 1 | 12 intervals in 24 hours | HPLC + LC-MS | 8 | no |
|  | 1990 [27] |  | 6 |  | T | H | 8w | 1 | 6, 10, 14, 18, 22 | HPLC | 7 | no |
|  | 2005 [66] |  | 200 | Healthy (n=136), LID (n=36), IDA (n=28) |  |  | 6m | 1 | 6, 12, 18, 00 |  | 5 | yes |
| *Vit B12* | 1994 [67] | Germany | 9 | PP + MP | T (38.5-40.9w) | F | 4-5d, 9-13d, 30-36d, 50-65d, 80-100d | 5x1 | Morning, midday, evening | Radioisotope analysis | 7 | no |
| *Cobalophi- lin* |  |  |  |  |  |  |  |  |  | Radioactive binding assay |  | no |
|  |  |  |  |  |  |  |  |  |  |  |  |  |
| Choline |  |  |  |  |  |  |  |  |  |  |  |  |
| *Choline* | 1986 [62] | USA (Massachusetts) | 5 |  |  | F+H | 39-85d | 72 hours | Each feeding for72 hours | RIA-like | 8 | no |
| *Phosphati- dylcholine* |  |  |  |  |  |  |  |  |  | RIA-like |  | no |
|  | Year | Location | N | Maternal  characteristics | Gestational  age at birth | Milk type | Postpartum   period | Collection   days | Collection  timepoints  (hours) | Analytical   method | RoB score | Circadian   pattern? |
| **Trace elements and elektrolytes** | | | | | | | | | | | | |
| Iron | 2005 [66] |  | 200 | Healthy (n=136), LID (n=36), IDA (n=28) |  |  | 6m | 1 | 6, 12, 18, 00 | reagent | 5 | yes, only in healthy |
|  | 1983 [69] | USA (Georgia) | 102 | PP + MP | T | F+Mid+H | 10-14d, 30-45d | 2x1 | 12-00, 00-12 | PES | 4 | yes |
|  | 1976 [51] | USA | 25 | PP + MP | P+T | F | 6-12w | 1 | 5-8, 12, 18 | SP | 7 | yes |
|  | 2000 [70] | Spain | 5 | PP + MP |  | F | 15d | 1 | 8, 22-24 | Flame AAS | 5 | yes |
| Sodium | 1982 [71] | USA (Texas) | 28 | PP + MP |  | F | 3.5-32w | 1 | 6-8, 10-12, 14-16, 18-20, 22-00, 2-4 | AES + AAS | 8 | yes |
|  | 2013 [35] | Brazil | 23 | Normotensive (n=10)^(A)^ hypertensive (n=13)^(B)^ |  |  | 3d, 10d, 23d | 3x1 | 12, 00 | SP | 5 | yes, C of (B) |
| Potassium | 1982 [71] | USA (Texas) | 28 | PP + MP |  | F | 3.5-32w | 1 | 6-8, 10-12, 14-16, 18-20, 22-00, 2-4 | AES + AAS | 8 | yes |
|  | 2013 [35] | Brazil | 23 | Normotensive (n=10)^(A)^ hypertensive (n=13)^(B)^ |  |  | 3d, 10d, 23d | 3x1 | 12, 00 | SP | 5 | yes, C of (B) |
| Calcium | 1983 [72] | USA (Georgia) | 102 | PP + MP | T | F+Mid+H | 10-14d, 30-45d | 2x1 | 12-00, 00-12 | PES | 4 | no |
|  | 1988 [73] | USA (Indiana) | 49 | PP + MP |  | F | 1m, 2m, 3m, 4m, 5m, 6m | 6x1 | 00-6, 6-10-, 10-14, 14-18, 18-21, 21-00 | AAS | 9 | no |
|  | 2013 [35] | Brazil | 23 | Normotensive (n=10), hypertensive (n=13) |  |  | 3d, 10d, 23d | 3x1 | 12, 00 | SP | 5 | no |
|  | 2007 [74] | Poland | 5 |  |  | H | 90-104d | 14x1 | 9, 15, 21 | AAS | 8 | no |
| Phospho- rus | 1983 [72] | USA (Georgia) | 102 | PP + MP | T | F+Mid+H | 10-14d, 30-45d | 2x1 | 12-00, 00-12 | PES | 4 | no |
|  | 2013 [35] | Brazil | 23 | Normotensive (n=10)^(A)^ hypertensive (n=13)^(B)^ |  |  | 3d, 10d, 23d | 3x1 | 12, 00 | SP | 5 | yes, M of (A) |
| Magnesi- um | 1983 [72] | USA (Georgia) | 102 | PP + MP | T | F+Mid+H | 10-14d, 30-45d | 2x1 | 12-00, 00-12 | PES | 4 | no |
|  | 1988 [73] | USA (Indiana) | 49 | PP + MP |  | F | 1m, 2m, 3m, 4m, 5m, 6m | 6x1 | 00-6, 6-10-, 10-14, 14-18, 18-21, 21-00 | AAS | 9 | yes |
|  | 2007 [74] | Poland | 5 | PP |  | H | 90-104d (14 consecutive days) | 14x1 | 9, 15, 21 | AAS | 7 | no |
| Copper | 1983 [69] | USA (Georgia) | 102 | PP + MP | T | F+Mid+H | 10-14d, 30-45d | 2x1 | 12-00, 00-12 | PES | 4 | no |
|  | 1976 [51] | USA | 25 | PP + MP | P+T | F | 6-12w | 1 | 5-8, 12, 18 | SP | 7 | yes |
|  | 2000 [70] | Spain | 5 | PP + MP |  | F | 15d | 1 | 8, 22-24 | Flame AAS | 5 | no |
|  | 2007 [74] | Poland | 5 | PP |  | H | 90-104d (14 consecutive days) | 14 | 9, 15, 21 |  | 8 | no |
| Zinc | 1983 [69] | USA (Georgia) | 102 | PP + MP | T | F+Mid+H | 10-14d, 30-45d | 2x1 | 12-00, 00-12 | PES | 4 | no |
|  | 1988 [73] | USA (Indiana) | 49 | PP + MP |  | F | 1m, 2m, 3m, 4m, 5m, 6m | 6x1 | 00-6, 6-10-, 10-14, 14-18, 18-21, 21-00 | AAS | 9 | yes |
|  | 1985 [76] |  | 16 |  |  | M | 1m, 2m, 3m, 4m, 5m, 6m, 7m, 8m, 9m, 10m, 11m, 12m | 12x1 | 6, 9, 12, 15, 18, 21 | AAS | 7 | no |
|  | 1976 [51] | USA | 25 | PP + MP | P+T | F | 6-12w | 1 | 5-8, 12, 18 | SP | 7 | yes |
|  | 2005 [75] | Egypt | 60 | PP, control (n=30), zinc supplement (n=30) | T, singletons |  | colostrum, 2m | 2x1 | 10, 22 | Flame SP | 6 | yes |
|  | 2000 [70] | Spain | 5 | PP + MP |  | F | 15d | 1 | 8, 22-24 | Flame AAS | 5 | no |
|  | 2007 [74] | Poland | 5 | PP |  | H | 90-104d | 14x1 | 9, 15, 21 | Voltam-metric | 8 | no |
| CuZn | 2010 [54] | Brazil | 36 | VD | T |  | 3d, 10d, 30d | 3x1 | 12, 00 | SP | 5 | yes, C |
| Iodide | 2007 [77] | USA | 8 |  |  |  |  | feb-14 | Before breakfast, after dinner | IC-MS | 3 | no |
| Iodine | 1960 [78] | Finland | 5 |  |  |  |  | 1 | 5x in 24 hours | Vilkki | 3 | no |
| Molybde- num | 1988 [79] |  | 7 |  | P (27-36w) +  T (38-41w) | M |  | 3-60 | 6-9, 9-12, 12-15, 15-18, 18-21, 21-00, 00-3, 3-6 | AAS | 4 | no |

***Bioactive factors***

|  | Year | Location | N | Maternal  characteristics | Gestational  age at birth | Milk type | Postpartum   period | Collection   days | Collection  timepoints  (hours) | Analytical   method | RoB score | Circadian   pattern? |
| --- | --- | --- | --- | --- | --- | --- | --- | --- | --- | --- | --- | --- |
| **Hormones** | | | | | | | | | | | | |
| Melatonin | 2012 [81] |  | 5 |  | P+T (33-42w) |  | 2-4m | 1 | 9, 11, 13, 15, 17, 19, 21, 23, 1, 3, 5, 7 | ELISA | 6 | yes |
|  | 2013 [82] | Brazil | 7 |  | T |  | 48-72h | 1 | 12, 00 | AC + ELISA | 4 | yes |
|  | 1993 [83] |  | 10 |  |  | H | 3-4d | 1 | 14-17, 2-4 | RIA | 5 | yes |
|  | 2016 [41] | Germany | 21 |  | P (27.6-36.1w) + T (37.0-39.4w) | H | 5-10d | 1 | 10-22, 22-10 | ELISA | 5 | yes |
|  | 2019 [84] | Israel | 10 |  | P+T |  | <6m | 1 | 3, 12 | ELISA | 3 | yes |
|  | 2007 [85] | Brazil | 24 | CS (n=9) +  VD (n=11) | >37w |  | 3d (n=24); 10d, 15d, 20d, 30d (n=4) | 5x1 | 12, 00 | ELISA | 7 | yes, VD |
|  | 2006 [86] | Brazil | 18 | Healthy (n=11), mastitis (n=8),  VD (n=11), forceps (n=8) | >37w |  | 48-72h | 1 | 12, 00 | ELISA | 7 | yes, only in healthy |
|  | 1981 [87] | Gambia | 60 | MP + PP |  | F+H | 1-18m | 1 | Each feeding 7-19h; for n=16 each feeding in 24 hours | CM | 8 | yes |
|  | 2013 [88] | Brazil | 42 |  | T (38.9±1.4w) |  | 3d, 10d, 30d | 3x1 | 12, 00 | ELISA | 6 | yes, C + T + M |
|  | 2019 [106] | China | 98 | CS (n=47) +  VD (n=51) | P+T (32.3-39.9w),  some with physiological jaundice | Mix | 0-7d, 8-14d, 15-30d | 3x1 | 3, 9, 15, 21 | ELISA | 8 | Yes |
| Cortisol | 2019 [89] | NL | 42 | 36 +/- 4.7y,  CS (n=21) + VD (n=21),  MP + PP | T (37-42w) | F | 30d (+/- 5d) | 1 |  | LC-MS | 9 |  |
|  | 2019 [24] | NL | 4 |  | T (37-42w) | F | 30d (+/- 5d) | 1 | 5x in 24 hours | LC-MS | 6 | yes |
|  | 1983 [90] | USA (Texas) | 19 | MP + PP |  | F |  | 1 | 6-8, 10-12, 22-00 | Competitive binding radioassay + RIA | 8 | yes |
|  | 2017 [91] | New Zealand | 23 |  | Corrected for parity | F+H | 3.2m | 1 | 4-10; 10-16; 16-22; 22-4 | LC-MS | 9 | yes |
|  | 2013 [88] | Brazil | 42 |  | T (38.9±1.4w) |  | 3d, 10d, 30d | 3x1 | 12, 00 | ELISA | 6 | yes, M |
|  | 2016 [92] | NL | 10 |  | T (37.1-41.3w) | F | 1m | 1 | 7-8x in 24 hours | LC-MS/MS | 10 | yes |
|  | 2019 [93] | NL | 54 | Psychopatology (n=15) | T (37-42w) | F | 1m (+/- 5d) | 1 | 12x in 24 hours | LC-MS/MS |  | yes |
| Cortisone | 2019 [89] | NL | 42 | CS (n=21) +  VD (n=21) | T (37-42w) | F | 30d (+/- 5d) | 1 |  | LC-MS | 9 |  |
|  | 2019 [24] | NL | 4 |  | T (37-42w), singletons | F | 30d (+/- 5d) | 1 | 5x in 24 hours | LC-MS | 6 | yes |
|  | 2016 [92] | NL | 10 |  | T (37.1-41.3w) | F | 1m | 1 | 7-8x in 24 hours | LC-MS/MS | 10 | yes |
|  | 2017 [91] | New Zealand | 23 | Corrected for parity |  | F+H | 3.2m | 1 | 4-10; 10-16; 16-22; 22-4 | LC-MS | 9 | yes |
|  | 2019 [93] | NL | 54 | Psychopatology (n=15) | T (37-42w), singletons | F | 1m (+/- 5d) | 1 | 12x in 24 hours | LC-MS/MS | 7 | yes |
| Leptin | 2015 [28] | Australia | 19 | MP + PP | T | F+H | 3-21w | 1 | 4-10, 10-16, 16-22, 22-4 | ELISA | 8 | yes |
| Prolactin | 2002 [94] | Australia | 20 | MP + PP | T | F+H | 1-19m | 1 | 22-2, 2-6, 6-10, 10-14, 14-18, 18-22 | IRMA | 9 | yes |
| PTHrP | 1998 [95] |  | 2 |  |  | F | 2w, 4w (n=1); 13w, 14w (n=1) | 2 (pooled) |  | IRMA | 5 | no |
|  | Year | Location | N | Maternal  characteristics | Gestational  age at birth | Milk type | Postpartum   period | Collection   days | Collection  timepoints  (hours) | Analytical   method | RoB score | Circadian   pattern? |
| **Immune factors** | | | | | | | | | | | | |
| Lactoferrin | 1983 [96] | USA (Georgia) | 89 |  | T | F+mid+H | 10-14d, 30-47d | 2x1 | Morning, evening | Immuno EP | 3 | no |
|  | 1985 [99] | Australia | 8 |  |  | F+H |  | 2 |  | ELISA | 4 | no |
| Immunoglobulins |  |  |  |  |  |  |  |  |  |  |  |  |
| *IgA* | 2010 [54] | Brazil | 36 | VD | T |  | 3d, 10d, 30d | 3x1 | 12, 00 | RID | 5 | yes |
| *IgM* |  |  |  |  |  |  |  |  |  | RID |  | yes, T + M |
| *IgG* |  |  |  |  |  |  |  |  |  | RID |  | yes, T + M |
| *IgA* | 1983 [96] | USA (Georgia) | 89 |  | T | F+mid+H | 10-14d, 30-47d | 2x1 | Morning, evening | ID | 3 | no |
| *IgG* |  |  |  |  |  |  |  |  |  | ID |  | no |
| *IgM* |  |  |  |  |  |  |  |  |  | ID |  | no |
| *IgA* | 2013 [35] | Brazil | 23 | Normotensive (n=10)^(A)^ hypertensive (n=13)^(B)^ |  |  | 3d, 10d, 30d | 3x1 | 12, 00 | Turbidimetric SP | 5 | yes, C + T + M of (A) |
| *IgG* |  |  |  |  |  |  |  |  |  | ID |  | no |
| *IgM* |  |  |  |  |  |  |  |  |  | ID |  | no |
| *IgA* | 1975 [97] |  | 1 |  |  | F+mid+H | 1-2m | 5 |  | ID | 4 | no |
| *IgG* |  |  |  |  |  |  |  |  |  | Turbidimetric SP |  | yes, C + T of (A) |
| *IgM* |  |  |  |  |  |  |  |  |  | Turbidimetric SP |  | yes, C of (A) + (B) |
| Complement factors |  |  |  |  |  |  |  |  |  |  |  |  |
| *C3* | 2010 [54] | Brazil | 36 | VD | T |  | 3d, 10d, 30d | 3x1 | 12, 00 | SP | 5 | yes |
| *C4* |  |  |  |  |  |  |  |  |  | SP |  | yes, T + M |
| Lysozyme | 1975 [97] |  | 1 |  |  | F+mid+H | 1-2m | 5 |  |  | 4 | no |
|  |  |  |  |  |  |  |  |  |  |  |  |  |
| Phagocytes | 2010 [54] | Brazil | 36 | VD | T |  | 3d, 10d, 30d | 3x1 | 12, 00 | SP | 5 | yes |
| **Cytokins** | | | | | | | | | | | | |
| Interleukins |  |  |  |  |  |  |  |  |  |  |  |  |
| *IL-2* | 2015 [98] | Brazil | 24 | SC | T |  | 3d, 30d | 2x1 |  | FC | 6 | no |
| *IL-4* |  |  |  |  |  |  |  |  |  | FC |  |  |
| *IL-6* |  |  |  |  |  |  |  |  |  | FC |  | yes, C |
| *IL-10* |  |  |  |  |  |  |  |  |  |  |  | no |
| *IL-17* |  |  |  |  |  |  |  |  |  | FC |  | no |
| *IL-2* | 2007 [85] | Brazil | 24 | CS (n=9) +  VD (n=11) | >37w |  | 3d (n=24); 10d, 15d, 20d, 30d (n=4) | 5x1 | 12, 00 | ELISA | 7 | yes, CS |
| *IL-4* |  |  |  |  |  |  |  |  |  | ELISA |  | yes |
| *IL-5* |  |  |  |  |  |  |  |  |  | ELISA |  | yes |
| *IL-10* |  |  |  |  |  |  |  |  |  | ELISA |  | yes |
| *IL-12* |  |  |  |  |  |  |  |  |  | ELISA |  | no |
| IFN-gamma | 2013 [35] | Brazil | 23 | Normotensive (n=10)^(A)^ hypertensive (n=13)^(B)^ |  |  | 3d, 10d, 23d | 3x1 | 12, 00 | ELISA | 5 | yes, T of (A) + (B) |
|  | 2015 [98] | Brazil | 24 | CS | T |  | 3d, 30d | 2x1 |  |  | 6 | no |
|  | 2007 [85] | Brazil | 24 | CS (n=9) +  VD (n=11) | >37w |  | 3d (n=24); 10d, 15d, 20d, 30d (n=4) | 5x1 | 12, 00 | ELISA | 7 | Yes, CS |
|  | 2013 [88] | Brazil | 42 |  |  |  | 3d, 10d, 30d | 3x1 | 12, 00 | ELISA | 6 | no |
| TGF-bèta | 2013 [35] | Brazil | 23 | Normotensive (n=10)^(A)^ hypertensive (n=13)^(B)^ |  |  | 3d, 10d, 23d | 3x1 | 12, 00 | ELISA | 5 | Yes, C in B |
|  | 2013 [88] | Brazil | 42 |  | T (38.9±1.4w) |  | 3d, 10d, 30d | 3x1 |  | ELISA | 6 | no |
| TNF-alfa | 2015 [98] | Brazil | 24 | CS | T |  | 3d, 30d | 2 |  | FC | 6 |  |
|  | 2007 [85] | Brazil | 24 | CS (n=9) +  VD (n=11) | T |  | 3d (n=24); 10d, 15d, 20d, 30d (n=4) | 5x1 | 12, 00 | ELISA | 7 | yes, VD |
|  | 2006 [86] | Brazil | 18 | Healthy (n=11),  mastitis (n=8),  VD (n=11), forceps (n=8) | T |  | 48-72h | 1 | 12, 00 | ELISA | 7 | yes, only in healthy |
| EGF | 1983 [100] | USA (Tennessee) | 8 | VD | P (<32w), singletons |  | 7-50d | 1 |  | RIA | 5 | yes, but wide intersubject variation |
|  | Year | Location | N | Maternal  characteristics | Gestational  age at birth | Milk type | Postpartum   period | Collection   days | Collection  timepoints  (hours) | Analytical   method | RoB score | Circadian   pattern? |
| **Antioxidants** | | | | | | | | | | | | |
| Anti-oxidant capacity | 2010 [103] | Spain | 7 |  |  | F | 1-5d | 1 | 00, 3, 6, 9, 12, 15, 18, 21 | SP | 7 | yes |
|  | 2016 [41] | Germany | 21 |  | P (27.6-36.1) + T (37.0-39.4) | H | 5-10d | 1 | 10-22, 22-10 | EA | 5 | no |
| Carotenoids |  |  |  |  |  |  |  |  |  |  |  |  |
| *Alpha carotene* | 1998 [104] |  | 7 | PP + MP | T | H | 9-16w | 1 | Morning, midday, evening | SP + HPLC | 6 | no |
| *Beta carotene* |  |  |  |  |  |  |  |  |  | SP + HPLC |  | no |
| *Lutein* |  |  |  |  |  |  |  |  |  | SP + HPLC |  | no |
| *Beta cryptoxanthin* |  |  |  |  |  |  |  |  |  | SP + HPLC |  | no |
| *Lycopene* |  |  |  |  |  |  |  |  |  | SP + HPLC |  | no |
|  | Year | Location | N | Maternal  characteristics | Gestational  age at birth | Milk type | Postpartum   period | Collection   days | Collection  timepoints  (hours) | Analytical   method | RoB score | Circadian   pattern? |
| **DNA/RNA** | | | | | | | | | | | | |
| MiRNAs |  |  |  |  |  |  |  |  |  |  |  |  |
| *MiR-21-5p* | 2015 [105] | France | 4 |  | P |  | 30-56d | 2-3 | 4-5x in 24 hours | qPCR + SP | 4 | no |
| *MiR-16-5p* |  |  |  |  |  |  |  |  |  | qPCR + SP |  | yes |
| *Let-7a-5p* |  |  |  |  |  |  |  |  |  | qPCR + SP |  | no |
| *Let-7g5p* |  |  |  |  |  |  |  |  |  | qPCR + SP |  | no |
| *Let-7d-5p* |  |  |  |  |  |  |  |  |  | qPCR + SP |  | no |
| *MiR-146b-5p* |  |  |  |  |  |  |  |  |  | qPCR + SP |  | no |
| Nucleotides |  |  |  |  |  |  |  |  |  |  |  |  |
| *5-AMP* | 2009 [101] | Spain | 30 |  |  | F | 3m | 1 |  | EP | 6 | yes |
| *5-GMP* |  |  |  |  |  |  |  |  |  | EP |  | yes |
| *5-CMP* |  |  |  |  |  |  |  |  |  | EP |  | yes |
| *5-IMP* |  |  |  |  |  |  |  |  |  | EP |  | yes |
| *5-UMP* |  |  |  |  |  |  |  |  |  | EP |  | no |
| *5-TMP* |  |  |  |  |  |  |  |  |  | EP |  | no |
| *AMP* | 1981 [102] |  | 6 |  |  |  |  | 1 |  | RIA | 3 | no |
| *GMP* |  |  |  |  |  |  |  |  |  | RIA |  | no |
|  | Year | Location | N | Maternal  characteristics | Gestational  age at birth | Milk type | Postpartum   period | Collection   days | Collection  timepoints  (hours) | Analytical   method | RoB score | Circadian   pattern? |
| **Other components** | | | | | | | | | | | | |
| Oligosa- charides  (HMO's) | 1986 [30] | France |  | PP + MP |  |  | 0-7d |  | 7,10,13,16,19,22 |  | 5 | no |
| Citrate | 1991 [26] |  | 12 |  |  | H | 1-13m | 1 | 8, 12, 16, 20, 24, 4 | EA | 6 | no |
| Malondi- aldehyde | 2005 [66] |  | 200 |  |  |  | 6m | 1 | 6, 12, 18, 00 |  | 5 | yes |
| Perchlo- rate | 2007 [77] | USA | 8 |  |  |  |  | 2-14 | Before breakfast, after dinner | IC-MS | 3 | yes |
| Thiocya- nate | 2007 [77] | USA | 8 |  |  |  |  | 2-14 | Before breakfast, after dinner | IC-MS | 3 | no |

**Supplemental File S4.** Overview of publications (n=83) reporting on circadian variation in human milk composition of macronutrients and micronutrients, including study characteristics, quality assessment and outcome of each study. Empty fields indicate that the information is not described in the reference. Articles are grouped by human milk component.
NL = The Netherlands, UK = United Kingdom, USA = United States of America, LID = Latent Iron Deficiency, IDA = Iron-deficiency anemia, CS = (only in ) caesarean section, VD = (only in) vaginal delivery, PP = primipara, MP = multipara, P = preterm birth, T = term birth, IRS = infrared spectroscopy, EA = enzymatic assay, SP = spectrophotometry, EP = elektrophoresis, KM = Kjeldahl method, RIA = radioimmunoassay, HPLC = high-performance liquid chromatography, CM = creamatocrit, TLC = thin-layer chromatography, AC = affinity chromatography, IRMA = immunoradiometric assay, RID = radial immunodiffusion, AC = affinity chromatography, PES = plasma emission spectrometry, AAS = atomic absorption spectrometry, AES = atomic emission spectrometry, IC = ion chromatography, F = Foremilk, H = Hindmilk, C = (only in) colostrum, T = (only in) transitional milk, M = (only in) mature milk.
